# Growth of alpine grassland will start and stop earlier under climate warming

**DOI:** 10.1038/s41467-022-35194-5

**Published:** 2022-12-01

**Authors:** Patrick Möhl, Raphael S. von Büren, Erika Hiltbrunner

**Affiliations:** Department of Environmental Sciences, University of Basel, Schönbeinstrasse 6, CH-4056 Basel, Switzerland

**Keywords:** Phenology, Ecophysiology, Plant ecology, Grassland ecology

## Abstract

Alpine plants have evolved a tight seasonal cycle of growth and senescence to cope with a short growing season. The potential growing season length (GSL) is increasing because of climate warming, possibly prolonging plant growth above- and belowground. We tested whether growth dynamics in typical alpine grassland are altered when the natural GSL (2–3 months) is experimentally advanced and thus, prolonged by 2–4 months. Additional summer months did not extend the growing period, as canopy browning started 34–41 days after the start of the season, even when GSL was more than doubled. Less than 10% of roots were produced during the added months, suggesting that root growth was as conservative as leaf growth. Few species showed a weak second greening under prolonged GSL, but not the dominant sedge. A longer growing season under future climate may therefore not extend growth in this widespread alpine community, but will foster species that follow a less strict phenology.

## Abstract (web summary)

Climate change and earlier snowmelt could potentially extend the growing season for alpine grassland plants. Here, the authors combine field and chamber controlled experiments to show that extending the summer period did not result in prolonged root and leaf growth.

## Introduction

In extratropical alpine environments, low temperature confines the growing season to 6–12 weeks^1^, forcing high-elevation plants to complete their annual developmental cycle within a short time. Yet, the duration of the growing season has increased considerably over the past decades due to above-average warming in mountain regions^2,3^, which has led to advanced snowmelt^4,5^. By the end of the century, snowmelt is expected to occur up to one month earlier in the Swiss Alps^5^ and autumn warming may further prolong the growing season length (GSL). Early release from snow cover commonly advances flowering phenology in many alpine species^6,7^, but less is known about how a longer growing season affects the temporal dynamics of growth and senescence^8,9^.

Remote-sensing studies highlighted that the greening of alpine plants tracks snowmelt within the current interannual variation^10,11^. When alpine vegetation responds to advanced snowmelt by growing earlier, the onset of senescence will determine how effectively the season is used for growth and resource acquisition^12^. However, leaf browning and senescence have received less attention in ecological studies than greening and growth^13^, and it is unclear how an early season start affects the onset of senescence in alpine grasslands. Early senescence in early starters may attenuate any growth-related effects in alpine and arctic vegetation^14–16^. And, if present, species-specific differences in the capability to delay senescence under favourable conditions may shape community composition in future.

Aboveground growth and tissue maintenance commonly stops early to prepare alpine plants for winter, while roots are better screened from first frost events in autumn and could therefore continue growing. Roughly two-thirds of the world’s grassland biomass is belowground^17^, and that fraction approaches 80–90% in arctic and alpine regions^1,18^. Despite the importance of roots and potential divergence between root and leaf phenology^19,20^, there is a lack of studies that explore the temporal dynamics of root growth in alpine grassland^21^. Unlike leaves, roots are hidden from remote sensing. Hence, our understanding of belowground processes relies entirely on local observations. Mini-rhizotrons are easily installed windows to examine root growth^22^ but processing the acquired images used to be extremely labour-intensive. Recently, machine learning algorithms have been developed that automatically distinguish between roots and soil in images^23^, allowing to analyze large datasets. Observations with high spatial or temporal resolution are needed to understand how above- and belowground phenology is linked^24,25^. This is crucial to understand current states and predict changes in alpine vegetation under climate warming.

**Fig. 1:**
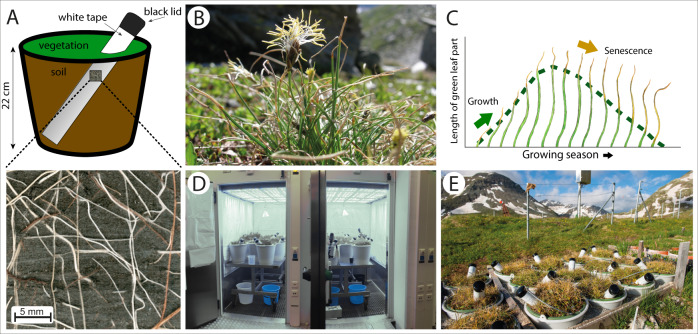
Overview of the experimental setup. **A** Scheme of a monolith with natural vegetation and its original soil, equipped with a transparent rhizotron tube to scan root growth. Roots grow along the tube surface (see insert below). **B** The dominant species *Carex curvula*. Photo: C. Körner. **C** Elongation and browning of a single *Carex* leaf in the course of a growing season. **D** Monoliths exposed to premature (+4 m, +2 m) summer conditions in climate chambers. **E** Monoliths at the alpine site during actual summer (July); note the advanced browning compared to the surrounding vegetation.

Here, we assessed whether alpine grassland is capable of extending growth and maintaining green tissues when subjected to a significantly longer GSL. We experimentally advanced the growing season by exposing monoliths of typical alpine grassland (*Caricetum curvulae*, Fig. 1) to typical summer conditions in climate chambers—two to four months before the actual growing season started. We combined repeated censuses of above- and belowground growth parameters throughout the prolonged season and quantified leaf growth in additional field microsites with varying snowmelt timing. We hypothesize that (1) the start and rate of growth are tracking the provided temperature conditions. We assume that (2) the onset of aboveground senescence depends on season start and plant species. Further, (3) we expect root growth to continue as long as soil temperatures are high enough. By combining new methods to analyze root phenology with robust aboveground measurements, our study offers insights into the controls of seasonal growth in alpine plant species.

## Results

### Aboveground growth

**Fig. 2:**
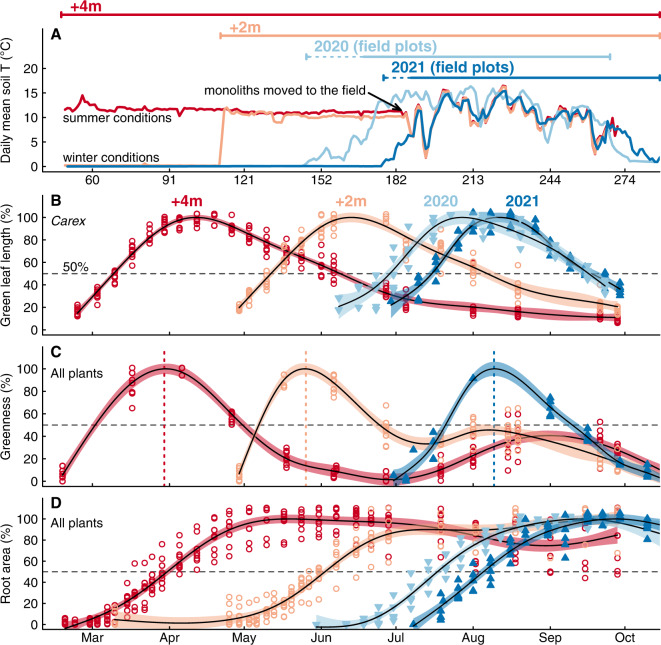
Impact of growing season length (GSL) on the timing of growth and senescence. Soil temperature and growth parameters with different growing season length, experimentally advanced in climate chambers (+4 m, +2 m, in 2021) and compared to field plots (2020, 2021). Day of the year is specified for the first day of each month below the *x* axis of **A**. GSL is indicated for each group at the top of **A** (dotted line during snowmelt). All growth data were scaled to 0–100% to ease comparison. **A** Daily mean soil temperature at 3–4 cm depth, close to the plants’ meristems. **B** Green leaf length of *Carex curvula*. **C** Canopy greenness of the whole plant community (2021). Dashed, vertical lines show the mean date for the peak. **D** Seasonal gain in root area per unit image area (mm^2^ cm^−2^, scaled to percent). Points indicate raw data and lines are GAM smoothers in **B**–**D** (lines: mean, error band: 95% confidence interval).

**Fig. 3:**
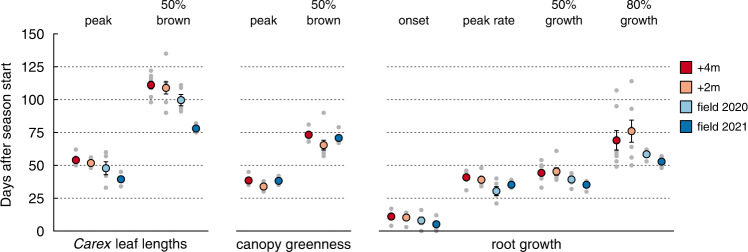
Timepoints related to growth and senescence for different growing season lengths (GSL). Peak green leaf length and senescence down to 50% browning for the dominant species *Carex curvula*, peak canopy greenness of the entire community and its decline to 50% and the onset of growth, highest growth rate, 50% and 80% seasonal growth for roots. GSL amounted to 238 d (+4 m), 174 d (+2 m), 109 d (field 2020) and 103 d (field 2021). Grey points show data for each monolith and field plot (8 monoliths for +4 m and +2 m and five field plots), colored points refer to mean ± SE (SE smaller than points are not visible).

We experimentally initiated the growing season in climate chambers, 70 and 134 days (termed ‘+2 m’ and ‘+4 m’, respectively) before the in-situ growing season started (Fig. 1, Table 1). Plants experienced similar environmental conditions in the climate chambers as in the field during summer (Fig. 2A), albeit with fixed diurnal conditions (see Methods section). Mean soil temperature during the first 50 days of the season amounted to 10.2 ± 0.1 °C in +4 m, 11.0 ± 0.1 °C in +2 m, and 10.7 ± 0.1 °C and 11.1 ± 0.1 °C in field plots of 2020 and 2021. Snowmelt in the field plots was 2021 around 3–4 weeks later than 2020 (earlier season start than usual). In both monolith groups and the field plots, leaf elongation of the dominant sedge *Carex curvula* All. s.str. (*Carex* hereafter) started right after the release from winter dormancy with exposure to temperatures >5 °C (Fig. 2B). It peaked after 44 d in field plots (mean of 2020/2021) and continued 9.3 ± 2.3 d longer in +4 m and +2 m (*t*_22_ = 4.1, *P* < 0.001), a brief extension only, given the substantial increase in GSL (Fig. 3). Peak leaf length averaged at 9.4 cm ± 0.4 and was not affected by GSL (*F*_3_ = 0.1, *P* = 0.93). Similar to leaf length, canopy greenness (assessed from photographs) increased right after the start of the season and peaked after 39 d in +4 m and field plots (no difference), but already after 34 d in +2 m (−4.5 ± 1.3 d, *t*_18_ = 3.4, *P* = 0.002, Fig. 2C). Hence, canopy greenness was obviously not reached later when exposed to earlier summer conditions.

**Table 1:** Characteristics of each experimental group (+4 m, +2 m, field plots) and microsites

	+4m	+2m	Field plots		Microsites
Year	2021	2021	2021	2020	2020
Sample size	8	8*	5	5	24
Size (m^2^)	0.06	0.06	0.32	0.32	0.16
Start of growing season	18 Feb	23 Apr	27 Jun–08 Jul	26 May–18 Jun	17 Mar–28 Jun
End of growing season^#^	15 Oct	15 Oct	15 Oct	25 Sep	25 Sep
Season length	238	174	99–110	99–122	89–192

*n = 7 for root measurements.

^#^End of meteorological growing season, caused by snowfall in 2020 and a cold spell in 2021.

Leaves of *Carex* brown from the tip towards the base (Fig. 1C), such that the remaining (decreasing) green leaf length reflects the progression of senescence. The time between peak leaf length of *Carex* and 50% leaf browning was 45 d in field plots and 11.7 ± 3.0 d longer in monoliths (*t*_22_ = 3.9, *P* < 0.001) with no difference between +4 m and +2 m. However, this difference was largely due to field plots in 2021, when browning took only 37 d compared to 52 d in 2020 (*t*_22_ = 3.2, *P* < 0.01, Fig. 3). Canopy greenness faded from 100% to 50% within 33 d, independent of GSL (*F*_2_ = 1.5, *P* = 0.24, Fig. 3). But unlike the monotonic leaf browning of *Carex*, the decline in canopy greenness of the entire community was partly reversible and greenness temporarily increased again by 11% in +2 m and 36% in +4 m later in the season (Fig. 2C). Although greenness peaked early, these very low values during the rest of the season accumulated to 49 ± 8.3% higher greenness (integrated as area under the curve) in monoliths than in field plots (*t*_18_ = 5.2, *P* < 0.001; no difference between +4 m and +2 m).

### Root growth

We observed root growth as increases in root area using mini-rhizotron tubes (Fig. 1A) and found that root growth started ca. 11 days after the onset of growing conditions in climate chambers (Fig. 3). Field plots of 2020 showed a similar delay as monoliths (8 days), but roots started 5.4 d earlier in the field in 2021 compared to monoliths (*t*_21_ = 2.4, *P* = 0.037). The majority of roots was produced within ca. two months after the start of the season: 80% of root growth was reached after 56 d in the field and after 73 d in +2 m and +4 m (Fig. 2D, Fig. 3). After that, root growth continued at a low rate, while +4 m even started to lose ca. −20% of its root area in the second half of the season (Fig. 2D). Thus, the experimentally added 134 d did not translate into sustained root growth in +4 m, and only 10% of root growth resulted from the additional 70 d in +2 m. Maximum increment rates were reached after 30–41 d, coinciding with peak canopy greenness (Fig. 2, Fig. 3). The total seasonal gain in root area was similar in all groups in 2021 (14–17 mm^2^ cm^2^), but significantly higher in the 2020 field plots (28 mm^2^ cm^2^; *t*_21_ = 4.7, *P* < 0.001). This is presumably related to the time since tube-installation (more unrooted space), as rooting had not yet reached steady-state. Overall, root diameters did not exceed 2.1 mm and averaged at 0.21 mm.

### Green cover and species-specific vigour index

**Fig. 4:**
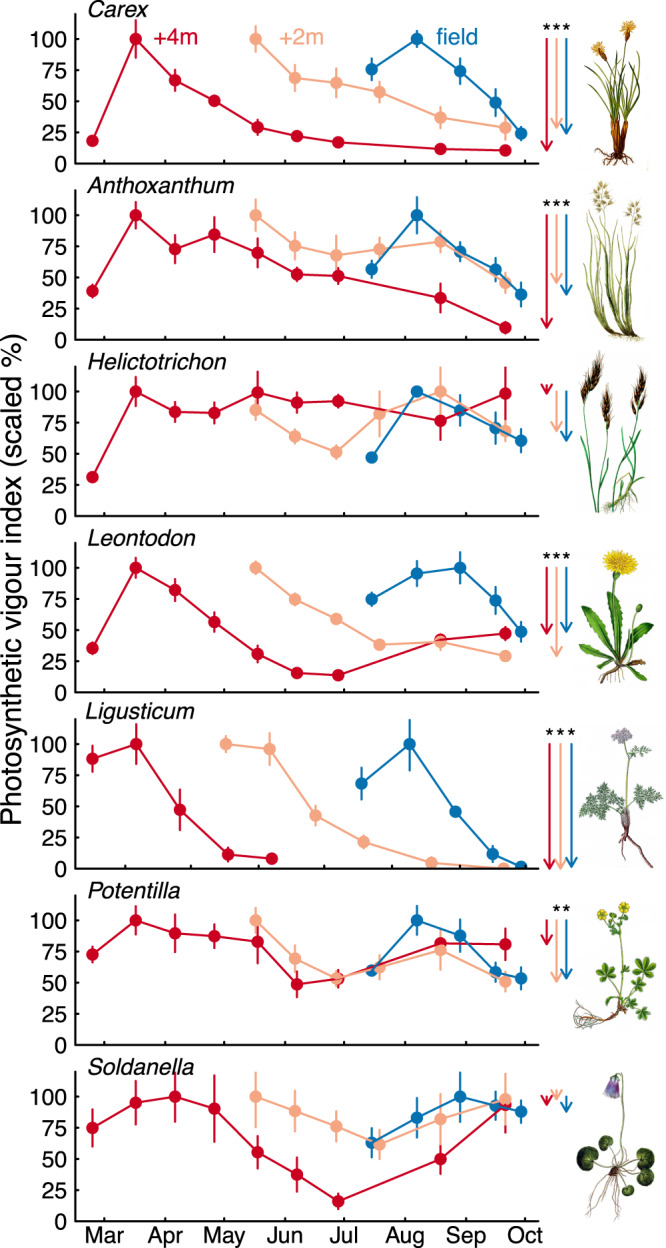
Maintenance of photosynthetically active tissue in the seven most abundant species over the season. Species-specific photosynthetic vigour index (mean ± SE) was calculated from number, size, green area, and chlorophyll content of leaves, in monoliths (+2 m, +4 m) and field plots. Data are scaled to percent of the maximum per species and group. Values were assessed for the same 1–3 individuals per experimental unit (8 monoliths for +4 m, +2 m, and 5 field plots) across the season. Arrows on the right side highlight the difference between the maximum and the last value of the season within the corresponding group. Asterisks indicate *P* < 0.05 (two-sided *t* tests, detailed statistics in Supplementary Table 2). Full species names are in Table 2. Illustrations provided by Oliver Tackenberg.

Total green plant cover decreased from ~65% during mid-season to <15% at the end of the season (Table 2, Supplementary Table 1). While green cover of all species was lower at the end of the season, some species lost more greenness compared to others. *Carex* was the dominant species during mid-season (28–37%), but made up only 1.4–12.7% of total green cover at the end of the season. Leaves of *Ligusticum* entirely disappeared within ca. 3 months, reducing green cover to zero. Green cover of *Anthoxanthum*, *Leontodon,* and *Potentilla* decreased to a similar degree as total green plant cover, leaving their relative contribution unchanged. In contrast, *Helictotrichon* and *Soldanella* constituted a 7% bigger fraction of the remaining green cover at the end of the season than during the mid-season (Table 2). Photosynthetic vigour index values (see Methods, Eq. 1) declined by 38–100% towards the end of the season in all species, except for the grass *Helictotrichon* (−24 ± 13%, *t*_9_ = 1.8, *P* = 0.16) and the forb *Soldanella* (−8 ± 15%, *t*_14_ = 0.5, *P* = 0.62; Fig. 4, Supplementary Table 2).

**Table 2:** Total green cover mid-season and at the end of the season and the contribution of the most abundant species (mean ± SE)

	+4 m		+2 m		Field plots		All groups	
	Mid-season	End-season	Mid-season	End-season	Mid-season	End-season	Δ(end - mid)	*P* value
Total green cover (%)	60.8 ± 2.8	13.3 ± 0.8	58.6 ± 4.2	11.0 ± 1.2	69.6 ± 2.6	8.1 ± 0.4	**−52.2 ± 2.2**	**<0.001**
Relative contribution (% of total)								
*Anthoxanthum alpinum* Á. & D. Löve	8.4 ± 2.1	18.8 ± 4.6	10.2 ± 3.7	14.9 ± 3.9	3.7 ± 0.9	2.2 ± 0.2	4.6 ± 2.9	0.130
*Carex curvula* All. s.str.	28.3 ± 3.7	1.4 ± 0.4	37.1 ± 5.0	3.8 ± 1.8	31.6 ± 1.3	12.7 ± 6.0	**−26.4 ± 3.0**	**<0.001**
*Helictotrichon versicolor* Vill.	4.0 ± 1.5	10.0 ± 3.5	4.1 ± 2.3	8.1 ± 4.3	10.2 ± 3.3	21.5 ± 7.1	**7.1 ± 3.0**	**0.020**
*Leontodon helveticus* Mérat	12.6 ± 2.6	8.3 ± 2.4	12.3 ± 1.6	11.0 ± 1.9	12.0 ± 2.2	3.7 ± 1.0	**−4.6 ± 1.8**	**0.010**
*Ligusticum mutellina* (L.) Crantz	7.7 ± 2.1	0.0 ± 0.0	5.9 ± 1.6	0.0 ± 0.0	5.5 ± 3.0	0.0 ± 0.0	**−6.3 ± 1.3**	**<0.001**
*Potentilla aurea* L.	11.9 ± 4.9	14.0 ± 4.3	5.2 ± 2.7	9.1 ± 3.8	9.1 ± 4.8	13.4 ± 5.7	3.4 ± 3.6	0.350
*Soldanella pusilla* Baumg.	1.7 ± 0.1	7.8 ± 0.6	2.4 ± 0.4	10.1 ± 1.4	1.2 ± 0.3	9.6 ± 2.4	**7.4 ± 0.8**	**<0.001**
Other species*	25.5 ± 3.6	39.7 ± 6.1	22.8 ± 5.4	43.0 ± 7.4	26.8 ± 4.9	37.0 ± 4.5	**14.9 ± 4.8**	**0.004**
Graminoids	45.7 ± 4.4	44.2 ± 5.6	61.8 ± 3.0	51.2 ± 6.1	47.2 ± 2.7	39.0 ± 5.2	−6.8 ± 4.1	0.109
Forbs	54.3 ± 4.4	55.8 ± 5.6	38.2 ± 3.0	48.9 ± 6.1	52.8 ± 2.7	61.0 ± 5.2	6.8 ± 4.1	0.107

Species cover was assessed 7–11 weeks after the start of the season (mid-season) and at the end of the season (19. October 2021). Differences between end- and mid-season across all groups are shown in the two last columns (statistically significant in bold, two-sided *t* tests). Detailed statistics are provided in Supplementary Table 1. *Alchemilla pentaphyllea L., *Geum montanum* L., *Gnaphalium supinum* L., *Homogyne alpina* (L.) Cass., *Leucanthemopsis alpina* (L.) Heywood s.str., *Nardus stricta* L., *Poa alpina* L., *Salix herbacea* L., *Sibbaldia procumbens* L., *Trifolium alpinum* L.

### Temperature effects in the field

**Fig. 5:**
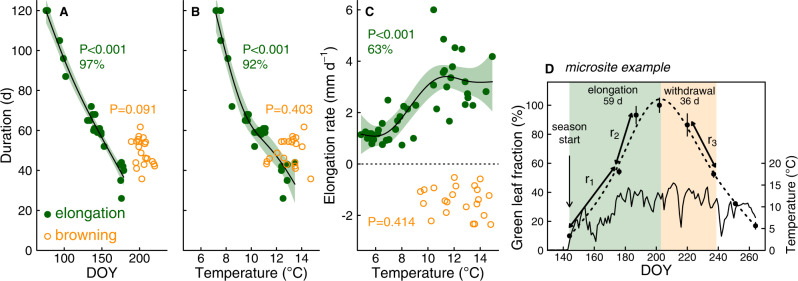
Duration and rates of growth and senescence in microsites (2020). Leaf elongation (green) and browning (orange) duration of *Carex curvula* related to **A** the onset of the respective period (*n* = 24 microsites for elongation and 20 for browning) and **B** to mean soil/meristem temperature (*n* = 23 for elongation and 20 for browning). **C** Daily rates of elongation and browning (negative) in relation to soil temperature (*n* = 43 measurement intervals for elongation and 22 for browning). **D** Exemplary data from one microsite illustrate how values in **A**–**C** were derived: elongation and browning period to 50% for **A** and **B**; rates (*r*_1–3_) for C, calculated for individual measurement intervals (mean ± SE, *n* = 5 leaves). Temperature was averaged over the corresponding periods. Smoothed curves (lines: mean, error band: 95% confidence interval) and variance explained (%) of smoothers are indicated only when smoothing terms were significant (*F* tests, *P* < 0.05, detailed statistics in Supplementary Table 3). DOY = day of year.

Due to low snow load and heavy storms in winter, snowmelt occurred exceptionally early at wind-exposed microsites in 2020. This led to substantial differences in snowmelt date between the 24 microsites (40 × 40 cm), where we monitored leaf elongation and browning in *Carex* (Table 1). Across microsites, leaf elongation until peak leaf length took longer under earlier snowmelt (*F*_2.2_ = 236.8, *P* < 0.001, Fig. 5A, Supplementary Table 3). As a consequence, the variation in snowmelt timing was considerably larger (103 days) than the resulting variation in the date of peak leaf length, which encompassed 31 days only. This variation in the leaf elongation period could be explained to 92% by soil temperature close to plants’ meristems (*F*_2.7_ = 79.1, *P* < 0.001, Fig. 5B), with faster elongation rates under warmer conditions (*F*_3.4_ = 17.1, *P* < 0.001, Fig. 5C). Nevertheless, peak leaf length (and the onset of browning) was reached 0.21 ± 0.04 days earlier per day of earlier snowmelt (*F*_1_ = 36.5, *P* < 0.001, *R*^2^ = 0.61). Leaf browning to 50% of maximum green length took 26 days and was independent of the date of peak leaf length (*F*_1_ = 3.2, *P* = 0.90) and soil temperature (*F*_1.6_ = 1.0, *P* = 0.40, Fig. 5C, Supplementary Figure 1). In contrast to experimental groups, maximum green leaf length varied across microsites but was not affected by snowmelt date or soil temperature (Supplementary Table 3).

## Discussion

We advanced the start of the alpine growing season and thus, pushed its total length to extremes: Our experiment more than doubled the available time for seasonal plant development and revealed an overarching autonomous control over growth and senescence. Whether the season was prolonged by two or four months, typical alpine summer conditions always initiated plant growth without major delay. However, early-onset of growth was accompanied by early-onset of senescence, halting above- and belowground plant growth even under ongoing, favourable summer conditions. Therefore, our findings challenge the widely assumed rise in future productivity as the thermal growing season prolongs due to climate warming.

A close correlation between snowmelt and the onset of leaf greening and elongation has previously been observed in alpine^26–28^ and arctic vegetation^29,30^. While climatic conditions for arctic and alpine plants differ in important aspects such as solar angle, photoperiod, precipitation and frost regime, they also share important similarities such as the short GSL^31^. The tight link between the start of growing conditions and actual growth substantiates that seasonally snow-covered plants leave endodormancy far ahead of actual snowmelt.

Nevertheless, it was speculated that an unusually short photoperiod may prevent growth in early spring^31^. But in contrast to flowering^6,7,32^, there is little evidence that vegetative growth of alpine plants is delayed by photoperiod in spring. We observed normal growth rates with a day-length of 14.5 h (1–1.5 months ahead of the natural season start) and previously even initiated typical spring growth using an 11.5 h-photoperiod for the same vegetation type (unpublished data). A study across ca. 25 alpine sites and 17 years found no indication that photoperiod influenced leaf elongation after snowmelt^28^. Beside its signalling effect, a short photoperiod also encompasses lower levels of photon fluxes, possibly limiting carbon uptake. However, perennial alpine plants have large belowground reserves^33^ and are not carbon-limited^34^, even under shade^35^.

Following snowmelt, temperature directly influenced the rates of leaf expansion and growth and thus, affected the time needed to reach peak leaf lengths (or maximum canopy greenness) and to enter leaf senescence. A correlation between leaf growth and temperature is well established from physiological studies in various plant species (e.g.,^36,37^), including alpine ones^38,39^. Low ambient temperatures are typical when snow melts earlier in the year, prolonging the required time to complete leaf elongation. Consequently, one day advance in snowmelt was associated with only 0.2 days earlier peak leaf length in our microsite survey. This is similar to observations from an interannual remote sensing study in the Swiss Alps, where peak NDVI of alpine grassland shifted by 0.5 days per day of earlier snowmelt^10^. In our experiment, leaf elongation did not take substantially longer in monoliths than in field plots, despite extremely advanced season start, most likely due to similar temperature after snowmelt. Hence, warmer spring temperatures under earlier snowmelt will enhance elongation rates until peak leaf lengths and advance the onset of senescence.

Given that senescence started after a similar timeframe in field plots and monoliths, the latter experienced a comparably long period with already senescing leaves. Moreover, the speed of leaf browning in *Carex* was 25% slower in monoliths compared to field plots. As leaf browning was equally slow between the two monolith groups, we do not anticipate that this difference between monoliths and field resulted from earlier snowmelt. Perhaps the maintained photoperiod or more stable temperature conditions could cause slower leaf browning. Temperature was not related to the speed of browning in our microsite survey, but a meta-analysis across 18 alpine and arctic sites of the International Tundra Experiment found that warming of 0.5–2.3 K significantly delayed leaf senescence by ca. 1 day^9^—a minor delay in relation to the projected advance in snowmelt^5^.

It seems that numerous alpine plants evolved conservative controls over senescence to guarantee completion of the seasonal development cycle within the short growing season^14,40,41^. To some degree, this is reflected in the annual biomass production: there is cumulative evidence that peak photosynthetic biomass (proxies like peak standing biomass, canopy height, or NDVI) of alpine grassland is independent of GSL and conserved across seasons^10,27,42–44^. We found that *Carex* reached the same maximum leaf lengths in all three experimental groups (+2 m, +4 m, and field plots). Apparently, seasonal biomass gain is shaped by other factors than GSL, such as temperature, water, and nutrient availability^1,45^.

Similar to leaves, root growth was initiated by the onset of growing conditions but postponed by several days. We assume that roots depend on aboveground signals to initiate growth, most likely mediated by hormones, such as auxin produced in young leaves^46^. A delay between the onset of above- and belowground growth was also observed in different arctic plant communities, where leaves always started growing prior to roots^19,29,47^. Delayed root growth in arctic regions could be a consequence of more prevalent soil frost that takes longer to melt—especially under lower solar angles. At least in alpine species, roots grew substantially less below 3–5 °C and ceased to grow in the range of 0.8–1.4 °C^39,48^.

In contrast to our hypothesis, root growth was not stimulated by extended summer conditions. After the initial growing phase of ca. 3 months, we found either no root growth or at a minute rate. Thus, both above- and belowground phenology were mostly completed after the duration that corresponds to a natural growing season. It seems that root growth stops once aboveground demands for nutrients and water decline. Or root growth is internally controlled, following similar phenological controls as observed in leaves^49^.

Compared to leaves, root senescence is difficult to document and requires chemical or molecular tools^50,51^. Color-changes such as browning in leaves are not a specific characteristic of senescing roots. Also, the visual distinction between dead and living roots is error-prone. Therefore, only roots that started to structurally disintegrate were considered dead, which was true for 0.3% of root area in the manually annotated mini-rhizotron images (see Methods). Such a low number of dead roots two years after the installation of the rhizotron tubes matches the commonly low root turnover rates of several years in alpine grassland^1,45^. Even fine roots may reach a substantial age of up to 15 years, as determined by mean residence time of carbon^52^, although carbon in roots may be older than the roots themselves^53,54^.

While all species responded opportunistically to a variable start of the season, most species were senescent during the long favourable second half of the season. The grass *Helictotrichon* and the snowbed plant *Soldanella* maintained high photosynthetic vigour index and made up a bigger fraction of the remaining green cover at end- compared to mid-season, indicating that these species could benefit from a longer season in terms of assimilation. In contrast, senescence of the dominant *Carex* progressed fast and deterministically. In the long run, species with such a conservative phenology may become outcompeted when a longer GSL ‘opens’ a window for additional growth during late season^16^. Yet, a 32-year monitoring study of the same grassland type reported only very small changes in species composition over time, despite climate warming and a probable increase in GSL^55^. The authors attributed this manifest stability of species composition to a lack of unoccupied sites in this densely rooted, late-successional grassland. Moreover, clonal proliferation is the rule in alpine grasslands and alpine species can be extremely persistent. In fact, individual clones of *Carex curvula* were found to live up to 5000 years^56^. Thus, species composition may remain stable for the coming decades or even centuries.

Our results provide experimental evidence that early snowmelt due to climate warming will trigger early senescence in this alpine vegetation type, both above- and belowground. Therefore, growth and carbon uptake do not scale with growing season length but strongly depend on internal controls that reflect an evolutionary adjustment to a short growing season. It came as a surprise that a 2–4 months earlier start resulted in a long period of senescent and brown vegetation during the second half of the growing season. This may lead to mismatches with soil microbial activities and therefore, with the nutrient cycle. Such a conservative control over seasonal development will constrain adjustments to the current pace of environmental changes, and in the longer term, promote species with a more flexible timing of growth and senescence.

## Methods

### Vegetation

The study was conducted on a *Caricetum curvulae* Br.-Bl., which is the most common alpine grassland community on acidic soils in the Alps^57^. This grassland is widespread in European alpine environments^58^ and shares traits with alpine sedge mats around the world (e.g., *Kobresia* grassland on the Tibetan Plateau), having a similar growth form, short stature, and persisting predominantly clonally. The sedge *Carex curvula* (Fig. 1B) is the dominant species, contributing around one third to total annual biomass production^35,59^. Grasses like *Helictotrichon versicolor* Vill. and forbs such as *Potentilla aurea* L. and *Leontodon helveticus* Mérat were also very abundant (Table 2). Leaves of *Carex* occur in tillers of 2–5 leaves that originate from belowground meristems. Every year, 1–2 (rarely 3) new leaves are formed that re-sprout in the following 2–3 years and then die off^59^. Growth and leaf elongation start rapidly after snowmelt (usually late June to early July) and reach a maximum before leaf senescence materializes as progressive browning from the leaf tip towards the base (Fig. 1C). By the end of season, the length of the green leaf part is reduced to 0.5–1.5 cm.

### Setup of the climate chamber experiment

In July 2019, we excavated 16 circular patches of homogenous vegetation (28 cm diameter, Fig. 1A) to a soil depth of ca. 22 cm, referred to as monoliths. They were collected in the vicinity of the ALPFOR research station at 2440 m a.s.l. in the Swiss Alps (46.577°N, 8.421°E) and fit into buckets with a perforated bottom to allow water to seep through (Fig. 1A). Soil and root systems of the monoliths were not further disrupted during that process. A transparent, acrylic rhizotron tube (inner diameter: 5.0 cm; outer: 5.6 cm) was installed in every monolith, protruding from the soil by ~15 cm (wrapped in a layer of black and white tape to block light and reduce heat absorption) and tilted at an angle of 35–45° to the surface (Fig. 1A). The lower opening of the tubes (in soil) was sealed with a rubber plug and the upper opening (outside of the soil) with a removable plastic cap. Polyethylene foam insulated the inside of the tubes.

During three summers, 2019–2021, the monoliths remained in sand beds in the natural, alpine environment next to the weather station of ALPFOR (Fig. 1E, www.alpfor.ch/weather.shtml). During alpine winter, monoliths were accessibly stored in a cold building at 1600 m elevation where monoliths were buffered from temperature fluctuations and screened from frost (Supplementary Figure 2). Monoliths were covered with cotton blankets and wooden boards to insulate plants, simulate snow pressure, and ensure complete darkness. This allowed a seamless transition to climate chambers before the start of the experiment, without exposing monoliths to freezing temperatures or sunlight. Monoliths had mean soil temperatures of 4.5 °C in the 2019/2020 winter and 3.5 °C in 2020/2021 (Oct–Feb, 3–4 cm soil depth, 3 HOBO TidBits, Onset Computer Corp., USA). In-situ, snow-covered soils rarely freeze due to the insulation by the snow pack and usually reach temperatures of around 0 °C. We do not expect that the slightly warmer soil affected temporal dynamics of plant growth, as roots and aboveground tissues remained visually dormant prior to the experiment. During a pilot study in April 2020, we exposed the monoliths to earlier summer conditions in climate chambers, but roots around the rhizotron tubes were not yet sufficiently established to permit root monitoring. Therefore, we postponed the experiment to 2021. Plants were moved to the climate chambers in February 2021, blankets still in place, and stored in the dark at 0 °C until the experiment started.

### Treatments

The 16 monoliths were equally distributed between two walk-in climate chambers (195 × 130 × 200 cm, L × W × H), in which temperature, light, humidity, and air circulation were controlled (Fig. 1D, phytotron facility^60^, University of Basel). Light was provided by 18 LED modules per chamber, comprising four separately dimmable light channels (blue [B], green, red [R], infrared [IR]; prototypes by DHL-Light, Hannover, GER). We took care to reach B:R ratios of natural sunlight on a bright day (ca. 0.8^61^) and set an R:FR ratio of ca. 1.4, which is above the range that characterizes vegetation shade. For summer conditions, photoperiod was set to 14.5 h, corresponding to early May in the central Alps (1–1.5 months prior to natural snowmelt), of which 12 h were at maximum light intensity (photon flux density of ca. 1000 μmol m^2^ s^−1^, Supplementary Figure 3). We set temperatures between 5 °C (night) and 14 °C (day) and logged soil temperature at 3–4 cm depth hourly throughout the experiment in six buckets per chamber (iButton DS1922L, Maxim Integrated Products Inc., USA).

Monoliths in the first chamber (termed ‘+4 m’) were exposed to alpine summer conditions on 18 February 2021, ~4 months before the in-situ start of the growing season. The second chamber remained dark at 0 °C until 23 April 2021, when the same summer settings were applied (‘+2 m’ group). Monoliths were watered twice a week with 0.8 L of deionized water per monolith. On 5 July 2021, all monoliths were transported to the alpine research site, experiencing natural growth conditions for the rest of the season. As a comparison, we studied five (untreated) plots of an already existing field experiment during two seasons (years 2020 and 2021), located at the same elevation 3 km away from the origin of the monoliths^7^. Each of these plots contained two rhizotron tubes within close proximity (30–40 cm apart; installed in July 2019). These in-situ plots became snow-free mid-June to early July and underwent natural growing seasons. As in +4 m and +2 m, soil temperature at 3-4 cm depth was logged once per hour in each field plot (HOBO TidBit, Onset Computer Corp., USA).

### Aboveground plant traits

For *Carex*, aboveground growth and senescence were assessed by measuring green leaf length from the soil surface to the narrow zone of incipient browning (similar to^27^). Each time, 5–10 leaves were randomly selected among the longest leaves. In +4 m, +2 m, and field plots, we measured 6–10, and in field microsites 5 leaves. To monitor the aboveground development of the entire community, we photographed the vegetation every 3–6 weeks in 2021 (DSLR D800, Nikon Corporation, JPN). From these images, we calculated canopy greenness to track temporal variation in plant phenology^62^: canopy greenness = G/(R + G + B), where R, G, and B represent the red, green, and blue channel, respectively. For leaf lengths and canopy greenness, the period of growth was defined as the time from the onset of summer conditions until the peak (100%) was reached. Senescence was defined as the period from the peak to 50% of leaf browning.

We obtained a proxy for the photosynthetically active leaf area of seven species (Table 2). Three individuals (in the case of graminoids: tillers) per monolith and plot were marked at the start of the growing season in 2021. Every 2–5 weeks, we assessed the number of intact leaves and the length of the longest leaf for each individual. Also, we estimated the fraction of brown leaf area compared to the total leaf area and measured leaf chlorophyll content by fluorescence ratio (emission ratio of intensity at 735 nm/700 nm) in the biggest, healthy-looking leaf (CCM-300, Opti-Sciences, Inc., USA). From these data, we calculated the following photosynthetic vigour index:

1 $${photosyn}{thetic}\,{vigour}\,{index}=	 \,{max}\,{leaf}\,{length}\,\times \,(1+\sqrt{{number}\,{of}\,{leaves}})\,\\ 	 \times \,(100\%\,-\,{brown}\,{leaf}\,{fraction})\,\\ 	 \times {chlorophyll}\,{content}$$

We used the square root of number of leaves to reflect the decrease in leaf size in each additional leaf beside the biggest leaf. To assess species-specific contributions to canopy greenness, green cover (0–100%) was estimated for each species two times: once during the season—after 11 weeks in +4 m and +2 m and after 7 weeks in field plots (in the field by eye)—and once at the end of the season (19 October 2021; from images).

### Root growth

We used two identical root scanners to produce high-resolution images (Fig. 1A, 1200 DPI) of roots growing along the surface of the tubes (CI-602, CID BioScience, USA). The scanner is inserted into the rhizotron tube to produce a 360°-image (21.6 × 18.6 cm, W × H) that is focused on the outer surface of the transparent tube (Fig. 1A). Each monolith and field plot were scanned throughout the growing season, twice a week during the first month and then at 7–21 days intervals. The average soil area and depth covered by the scans amounted to 330 cm^2^ and 18 cm per tube, respectively.

Root images were processed using Python 3 (v. 3.6.9). Vertical striping artifacts, frequent with such scanners, were removed^63^ and the aboveground part of the images (sun-block tape) was replaced by black. Brightness and contrast were normalized for each image before all images per tube were aligned (planar shifts determined by phase correlation). In total, we acquired ~700 scans and each was split into 16 sub-images measuring 2550 × 2196 pixels. Two sub-images per monolith/plot (one of each tube in field plots) were randomly chosen for manual root annotation using the rhizoTrak^64^ plugin (v. 1.3) for Fiji^65^. Of these 42 annotated images, half were used for training and half for validation of a convolutional neural network^66^. The training dataset was augmented with annotated images from another experiment at the site of the field plots (50 additional images, same size). Validation was performed on images from this study only. After 60 training epochs (i.e., training cycles through the entire dataset), 84% of all pixels predicted as root actually belonged to roots and 82% of the actual root pixels were identified as such. Subsequently, all original (full-sized) images were automatically segmented. Mean root area per image area (mm^2^ cm^−2^) was determined using RhizoVision^67^ (v. 2.0.3). Predicted root area correlated well with the actual root area in the manually annotated images (*R*^2^ = 0.99, Supplementary Figure 4). Dead-looking roots were found in 15 annotated images (0.3% of the total root area). Root data from one monolith was excluded because roots at the tube surface were scarce for unknown reasons.

### Microsites in the field

We chose 24 microsites (40 × 40 cm) covering different snowmelt dates and tracked leaf elongation and browning of *Carex*. Microsites were situated within an area of ~3 km^2^ around the research station (2283–2595 m a.s.l.) and were visited at irregular intervals during the growing season 2020. When microsites were measured twice within the same week (interval < 7 days), data were pooled and assigned to the mean date to reduce noise in the data. Each microsite was measured 5–10 times across the growing season (for an example, see Fig. 4D). As we suspected temperature to be a major driver of plant growth, and to determine the exact snowmelt-date, temperature sensors (iButton DS1922L) were installed 3 cm below the soil surface (close to *Carex*’s meristems) in each microsite in September 2019, logging temperature every two hours until the end of the growing season 2020.

### Data analysis

Data analyses were performed using the statistical programming language R^68^ (v. 4.0.5). To ease comparability between temporal sequences of response variables, *Carex* leaf length, canopy greenness, root area and photosynthetic vigour index were scaled to percent of the maximum (0–100%) for each group and species. Further, root area was set to zero at the start of the season. We fitted generalized additive models (GAM, mgcv-package^69^) with a thin-plate smoothing spline in the form ‘response variable ~ s(day of year)’ for each experimental unit. Number of knots (k) depended on sample size but was restricted to a maximum of eight and the estimated degrees of freedom varied between 3.1 and 6.9. Goodness of fit of smoothed terms was high in all cases (mean *R*^2^ > 0.88 for each response variable). The timepoints presented in Fig. 3 (e.g., 80% quantile of root growth) were interpolated using these GAMs, except for the day of 50% browning in green leaf length and greenness, which was linearly interpolated amid the closest measurements. Integrated area under the smoothed curve was approximated on a daily interval for greenness. The start of root growth was defined as the first date of a moving window, spanning three adjacent measurement dates, whose linear regression slope exceeded 0.5% d^−1^. Means and standard errors (SE) were calculated for each group (*n* = 7–8 in +4 m and +2 m, *n* = 5 in field plots). For visual simplicity, one GAM was fitted per group in Fig. 2.

For microsites, green leaf length of *Carex* was fixed at 0.5 cm at season start, which is about the amount of remaining green leaf previously observed after winter. Elongation and browning rates in microsites were calculated between consecutive measurements from season start to two weeks before the peak and from two weeks after the peak until one week following 50% browning, excluding the peak with intrinsically low rates. This yielded 43 elongation and 22 browning rates with intervals between measurements of 7–89 days. Corresponding mean soil temperature and growing degree hours (GDH) > 5 °C at 3 cm soil depth were calculated for each interval per microsite. Four microsites were not measured after 50% browning and were excluded from the analysis of browning periods. Also, one elongation period could not be related to temperature due to T-sensor failure.

The start of the growing season was defined as the day when snow disappeared, indicated by soil temperatures >3 °C and diurnal temperature fluctuations. Significant snowfall on 25 September in 2020 and a cold spell after 15 October in 2021 marked the meteorological end of the growing seasons for all plots. Differences between treatments were calculated by fitting linear regressions and subsequently calculating post-hoc contrasts using the R-package ‘emmeans’^70^. Model assumptions regarding residual distribution were verified visually. In the case of photosynthetic vigour index, maximum and last values were compared by fitting mixed effect models to account for repeated measures (package nlme). *P* values as well as *F* or *t* values with degrees of freedom based on the overall model are reported in text and in Supplementary Tables.

### Reporting summary

Further information on research design is available in the Nature Portfolio Reporting Summary linked to this article.

## Supplementary information

Supplementary Information

Reporting Summary

## Data Availability

Data generated in this study and annotated images used to train the neural network have been deposited in the figshare repository under accession code 10.6084/m9.figshare.20440497^71^.

## Code availability

R-codes are published with the data.
