# Supplementary material for: Growth of alpine grassland will start and stop earlier under climate warming
Source: Nat Commun. 2022 Dec 1;13:7398. doi: 10.1038/s41467-022-35194-5 (PMC9715633; doi:10.1038/s41467-022-35194-5)
Supplement: Supplementary file 1 — Supplementary Information [file 41467_2022_35194_MOESM1_ESM.pdf]

## Supplementary Tables

Supplementary Table 1: Detailed statistics for Table 2 of the main text. Post-hoc contrasts were performed using the emmeans-package for R (see Methods). P-values refer to two-sided t-tests without adjustment for multiple testing, as tests were performed to assess individual contribution rather than overall significance.

| Species | Δ(end - mid) | df | t | *P*-value |
|---|---|---|---|---|
| Total | -52.2 ± 2.2 | 20.8 | 24.2 | <0.001 |
| Relative contribution (% of total) | | | | |
| *Anthoxanthum alpinum* Á. & D. Löve | 4.6 ± 2.9 | 36 | -1.6 | 0.130 |
| *Carex curvula* All. s.str. | -26.4 ± 3.0 | 36 | 8.9 | <0.001 |
| *Helictotrichon versicolor* Vill. | 7.1 ± 3.0 | 36 | -2.3 | 0.020 |
| *Leontodon helveticus* Mérat | -4.6 ± 1.8 | 36 | 2.6 | 0.010 |
| *Ligusticum mutellina* (L.) Crantz | -6.3 ± 1.3 | 36 | -0.9 | <0.001 |
| *Potentilla aurea* L. | 3.4 ± 3.6 | 36 | 5.0 | 0.350 |
| *Soldanella pusilla* Baumg. | 7.4 ± 0.8 | 36 | -8.9 | <0.001 |
| Other species* | 14.9 ± 4.8 | 36 | -3.1 | 0.004 |
| Graminoids | -6.8 ± 4.1 | 36 | 1.6 | 0.109 |
| Forbs | 6.8 ± 4.1 | 36 | -1.7 | 0.107 |

* *Alchemilla pentaphyllea* L., *Geum montanum* L., *Gnaphalium supinum* L., *Homogyne alpina* (L.) Cass., *Leucanthemopsis alpina* (L.) Heywood s.str., *Nardus stricta* L., *Poa alpina* L., *Salix herbacea* L., *Sibbaldia procumbens* L., *Trifolium alpinum* L.

Supplementary Table 2: Percentage decrease in photosynthetic vigour index between the maximum and the last value of the season. Temporal development of photosynthetic vigour index is shown in Figure 4 of the main text. Post-hoc contrasts (two-sided t-tests) were performed using the emmeans-package for R (see Methods).

| Species | Group | Δ(max - last) | df | t | P |
|---|---|---|---|---|---|
| ***Anthoxanthum*** | All | 69 ± 8 | 16 | 8.2 | <0.001 |
| | +4m | 90 ± 14 | 16 | 6.6 | <0.001 |
| | +2m | 54 ± 14 | 16 | 4 | 0.001 |
| | Field plots | 64 ± 16 | 16 | 3.9 | 0.001 |
| ***Carex*** | All | 79 ± 7 | 18 | 10.6 | <0.001 |
| | +4m | 90 ± 12 | 18 | 7.6 | <0.001 |
| | +2m | 71 ± 12 | 18 | 6.1 | <0.001 |
| | Field plots | 76 ± 15 | 18 | 5.1 | <0.001 |
| ***Helictotrichon*** | All | 24 ± 13 | 9 | 1.8 | 0.100 |
| | +4m | 1 ± 22 | 9 | 0 | 0.968 |
| | +2m | 32 ± 26 | 9 | 1.2 | 0.250 |
| | Field plots | 39 ± 20 | 9 | 2 | 0.080 |
| ***Leontodon*** | All | 58 ± 4 | 18 | 14.3 | <0.001 |
| | +4m | 53 ± 6 | 18 | 8.2 | <0.001 |
| | +2m | 71 ± 6 | 18 | 11 | <0.001 |
| | Field plots | 51 ± 8 | 18 | 6.3 | <0.001 |
| ***Ligusticum**** | All | 100 ± 8 | 8 | 12 | <0.001 |
| | +4m | 100 ± 12 | 8 | 8.2 | <0.001 |
| | +2m | 100 ± 11 | 8 | 8.8 | <0.001 |
| | Field plots | 100 ± 19 | 8 | 5.4 | 0.001 |
| ***Potentilla*** | All | 38 ± 6 | 11 | 6.1 | <0.001 |
| | +4m | 19 ± 10 | 11 | 1.8 | 0.094 |
| | +2m | 49 ± 10 | 11 | 4.7 | 0.001 |
| | Field plots | 47 ± 12 | 11 | 4 | 0.002 |
| ***Soldanella*** | All | 8 ± 15 | 14 | 0.5 | 0.617 |
| | +4m | 9 ± 23 | 14 | 0.4 | 0.685 |
| | +2m | 2 ± 25 | 14 | 0.1 | 0.936 |
| | Field plots | 12 ± 31 | 14 | 0.4 | 0.703 |

*one-sided t-tests against zero because all leaves died before the end of the season

Supplementary Table 3: Generalized additive models (GAM) for *Carex* leaf elongation and browning at microsites. A thin plate regression spline smoother of k = 4 was assigned to predictor variables. edf = estimated degrees of freedom; DE = deviance explained; MLL = maximum leaf length. Significant variables are presented in bold.

| | | | Smoother | | | | |
|---|---|---|---|---|---|---|---|
| | Response (Y) | Predictor (X) | n | edf | *P*-value | $R^2$ | DE |
| Elongation | **Duration (d)** | **Season start (DOY)** | **24** | **2.2** | **<0.001** | **0.96** | **97%** |
| | **Duration (d)** | **Mean temperature (°C)** | **23** | **2.7** | **<0.001** | **0.91** | **92%** |
| | **Rate (mm $d^{-1}$)** | **Mean temperature (°C)** | **43** | **3.4** | **<0.001** | **0.60** | **63%** |
| | MLL (cm) | Duration (d) | 24 | 1 | 0.08 | 0.09 | 13% |
| | MLL (cm) | Mean temperature (°C) | 23 | 1 | 0.06 | 0.12 | 16% |
| Browning | Duration (d) | Peak leaf length (DOY) | 20 | 1 | 0.09 | 0.10 | 15% |
| | Duration (d) | Mean temperature (°C) | 20 | 1.7 | 0.40 | 0.05 | 14% |
| | Rate (mm $d^{-1}$) | Mean temperature (°C) | 22 | 1 | 0.41 | 0.00 | 3% |
| | MLL (cm) | Duration (d) | 20 | 1.9 | 0.13 | 0.19 | 27% |
| | MLL (cm) | Mean temperature (°C) | 20 | 1 | 0.37 | 0.00 | 5% |

## Supplementary Figures

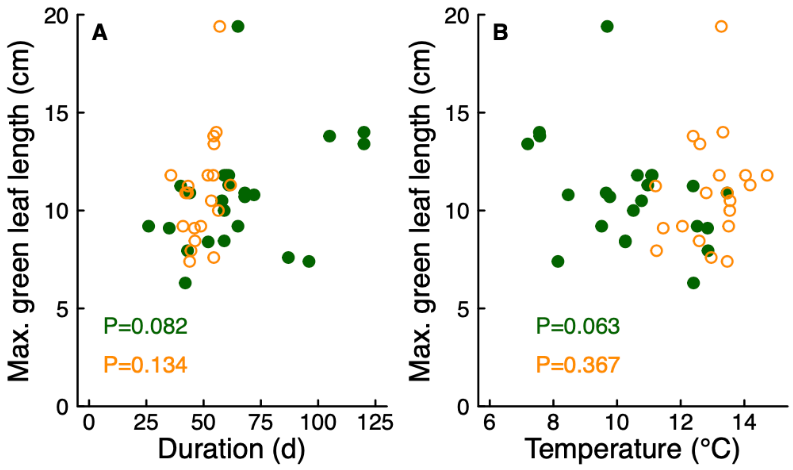


Supplementary Figure 1: Maximum green leaf lengths of *Carex curvula* in microsites. **A)** Maximum green leaf length was unrelated to the duration of leaf elongation (green) and browning (orange, open circles) in *Carex* at microsites. **B)** Maximum leaf length was also unrelated to soil temperature during the periods of elongation and browning. P-values were obtained from F-tests.

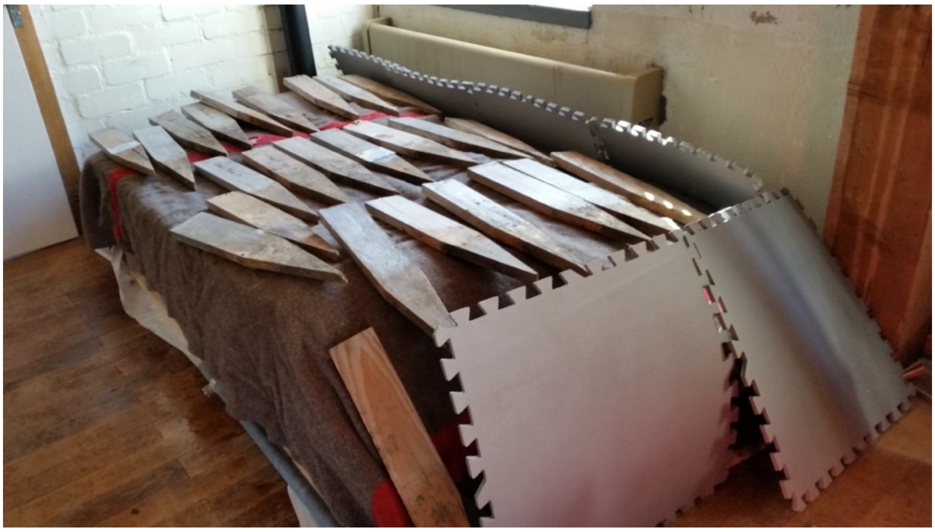

Supplementary Figure 2: Overwintering of monoliths in a cold house prior to transferring them to climate chambers. Blankets and wooden boards simulate the load of a snowpack. Digging out monoliths from snow during winter would expose them to unwanted freezing temperatures.

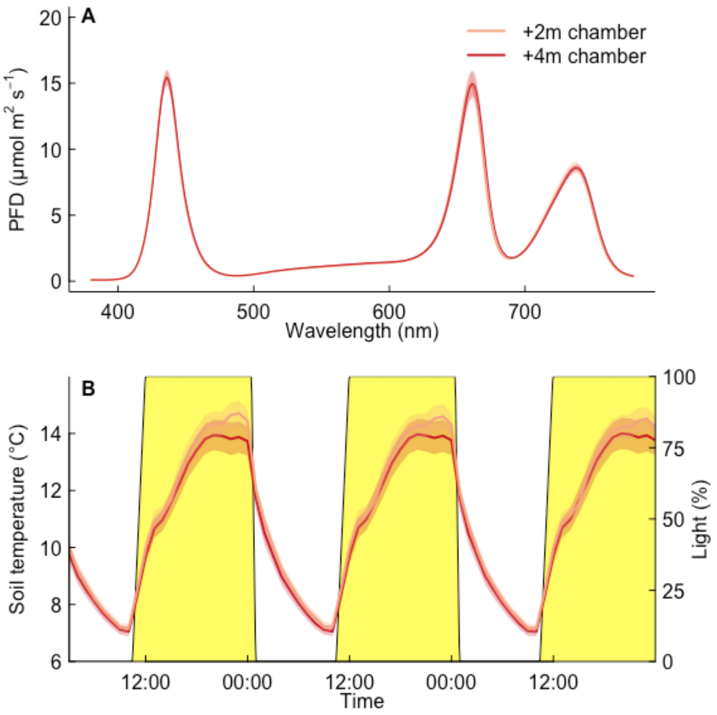


Supplementary Figure 3: Environmental conditions in the two climate chambers. **A)** Light spectra. The two curves (red and orange) overlap to a high degree. **B)** Diurnal pattern of soil temperature (red, measured at 3.5 cm soil depth in monoliths) and light (yellow) for three exemplary days in the climate chambers (daily average of 11 °C). Lines and error bands indicate means ± SE (n = 8 technical replicates in panel A, n = 6 monoliths in panel B for each chamber).

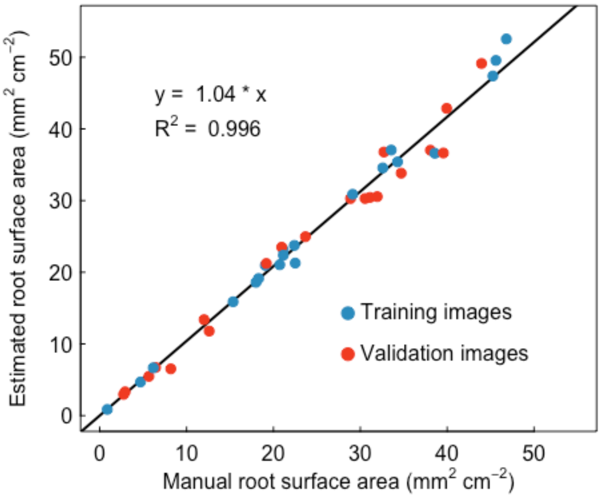


Supplementary Figure 4: Comparison between estimated and manually measured root surface area. Estimated root surface area in 40 automatically segmented images (2550 x 2196 px) that were previously segmented manually. Manual root area was exported from RhizoTrak (v 1.3). Two images (of 42) were not included, because manual root area was not available. Estimated root area was acquired using the software RhizoVision (v 2.0.3) on binary images (roots black, background white) generated by the convolutional neural network.
