# Supplementary material for: Growth of alpine grassland will start and stop earlier under climate warming
Source: Nat Commun. 2022 Dec 1;13:7398. doi: 10.1038/s41467-022-35194-5 (PMC9715633; doi:10.1038/s41467-022-35194-5)
Supplement: Supplementary file 2 — Reporting Summary [file 41467_2022_35194_MOESM2_ESM.pdf]

nature portfolio

Corresponding author(s): Patrick Möhl

Last updated by author(s): Sep 29, 2022

Reporting Summary

Nature Portfolio wishes to improve the reproducibility of the work that we publish. This form provides structure for consistency and transparency in reporting. For further information on Nature Portfolio policies, see our Editorial Policies and the Editorial Policy Checklist.

Statistics

For all statistical analyses, confirm that the following items are present in the figure legend, table legend, main text, or Methods section.

n/a	Confirmed	
☐	☒	The exact sample size (*n*) for each experimental group/condition, given as a discrete number and unit of measurement
☐	☒	A statement on whether measurements were taken from distinct samples or whether the same sample was measured repeatedly
☐	☒	The statistical test(s) used AND whether they are one- or two-sided<br>*Only common tests should be described solely by name; describe more complex techniques in the Methods section.*
☐	☒	A description of all covariates tested
☐	☒	A description of any assumptions or corrections, such as tests of normality and adjustment for multiple comparisons
☐	☒	A full description of the statistical parameters including central tendency (e.g. means) or other basic estimates (e.g. regression coefficient) AND variation (e.g. standard deviation) or associated estimates of uncertainty (e.g. confidence intervals)
☐	☒	For null hypothesis testing, the test statistic (e.g. *F*, *t*, *r*) with confidence intervals, effect sizes, degrees of freedom and *P* value noted<br>*Give P values as exact values whenever suitable.*
☒	☐	For Bayesian analysis, information on the choice of priors and Markov chain Monte Carlo settings
☒	☐	For hierarchical and complex designs, identification of the appropriate level for tests and full reporting of outcomes
☒	☐	Estimates of effect sizes (e.g. Cohen's *d*, Pearson's *r*), indicating how they were calculated

Our web collection on statistics for biologists contains articles on many of the points above.

Software and code

Policy information about availability of computer code

Data collection	Neural network published as standalone code in Smith et al. (2020) (https://github.com/Abe404/segmentation_of_roots_in_soil_with_unet)<br>Fiji v. 1.0 (https://imagej.net/software/fiji/)<br>RhizoTrak (Fiji-Plugin) v. 1.3 to annotate root images (https://prbio-hub.github.io/rhizoTrak/)<br>All references are provided in the manuscript
Data analysis	Statistical programming language R (R Core Team, 2021, v. 4.05)<br>Python programming language (v. 3.6.9)<br>RhizoVision by Seethepalli et al. (2020) to analyse segmented root images (v2.0.3, https://www.rhizovision.com/)

For manuscripts utilizing custom algorithms or software that are central to the research but not yet described in published literature, software must be made available to editors and reviewers. We strongly encourage code deposition in a community repository (e.g. GitHub). See the Nature Portfolio guidelines for submitting code & software for further information.

Data

Policy information about availability of data

All manuscripts must include a data availability statement. This statement should provide the following information, where applicable:

- Accession codes, unique identifiers, or web links for publicly available datasets
- A description of any restrictions on data availability
- For clinical datasets or third party data, please ensure that the statement adheres to our policy

Data generated in this study and annotated images used to train the neural network have been deposited in the figshare repository under accession code https://doi.org/10.6084/m9.figshare.20440497.

Human research participants

Policy information about studies involving human research participants and Sex and Gender in Research.

Reporting on sex and gender	N/A
Population characteristics	N/A
Recruitment	N/A
Ethics oversight	N/A

Note that full information on the approval of the study protocol must also be provided in the manuscript.

Field-specific reporting

Please select the one below that is the best fit for your research. If you are not sure, read the appropriate sections before making your selection.

☐ Life sciences ☐ Behavioural & social sciences ☒ Ecological, evolutionary & environmental sciences

For a reference copy of the document with all sections, see nature.com/documents/nr-reporting-summary-flat.pdf

Ecological, evolutionary & environmental sciences study design

All studies must disclose on these points even when the disclosure is negative.

Study description	We transferred intact vegetation including soil into buckets ('monoliths') and exposed them to a considerably earlier season start in climate chambers in 2021. Once actual snowmelt occurred at the site of origin, these monoliths were brought to the field and remained there until the end of the season. As a comparison, we studied five plots of the same vegetation (alpine grassland) in the field for two years (2020 and 2021). We repeatedly measured leaf elongation and browning of the dominant sedge, canopy greenness, root growth (rhizotron tubes), and a photosynthetic vigour index in the seven most abundant species to assess the temporal development of these traits under a longer growing season. To investigate temperature effects on leaf elongation and browning of the dominant sedge, we studied 24 microsites with differing snowmelt timing in the field throughout one season.
Research sample	We studied alpine grassland of the the type Caricetea curvulae Br.-Bl. (see e.g. Landolt 2012), which is dominated by the sedge Carex curvula. We chose this vegetation type because it is the most common alpine grassland on acidic soil in the Alps and forms closed, rather homogeneous communities. Our samples reflect the entire community of plants (each monolith and plot contained ca. 5–15 different species), including neighboring species and intact soil and root interactions.
Sampling strategy	We chose a comparably high sample size of 8 for each of the two monolith groups to make sure that installation effects and slight differences in species composition between monoliths will not affect our outcome. The size of each monolith (28 cm diameter and ca. 22 cm deep) was chosen as big as possible while keeping the weight in a range that allows manual handling. In the field, we studied 5 control plots from an existing field experiment of the same vegetation type. This lower sample size was justified because (1) plants remained in the field and (2) we measured these plots for two instead of only one year. Our data showed that both sample sizes were sufficient for the scope of this study. Green leaf lengths were asessed with a ruler, measuring from soil surface to the green tip. Greenness, i.e. the green fraction of an image, was extracted from photographs taken with a digital camera using Python. Root growth was assessed with rhizotron images that were analyzed using a convolutional neural network. Root area was extracted from binary images (white: roots, black:soil) using RhizoVision. To calculate photosynthetic vigour indices, we determined number of leaves, measured the biggest leaf (ruler), estimated the fraction of brown leaf area by eye and measured chlorophyll content by fluorescence ratio in individual plants.
Data collection	Soil temperature was measured with temperature loggers in all treatment groups as well as in microsites. Green leaf lengths of Carex curvula were measured with a ruler repeatedly during the season. To monitor the development of canopy greenness of the entire

community, we photographed the vegetation every 3–6 weeks in 2021. Rhizotron tubes were scanned throughout the growing season, twice a week during the first month and then at 7–21 days intervals. Three individuals (in the case of graminoids: tillers) per monolith and plot were marked at the start of the growing season in 2021. Every 2–5 weeks, we assessed the number of intact leaves and the length of the longest leaf for each individual. Also, we estimated the fraction of brown leaf area compared to the total leaf area and measured leaf chlorophyll content by fluorescence ratio (emission ratio of intensity at 735nm / 700nm) in the biggest, healthy-looking leaf. From these data, we calculated the following photosynthetic vigour index.

Microsites were visited repeatedly during the season to measure green leaf lengths of the dominant sedge Carex curvula with a ruler.

Patrick Möhl collected data on the main experiment and Raphael S. von Büren collected data in the separate microsites.

Timing and spatial scale	Monoliths were excavated in 2019 and the experiment took place in 2021. Control plots were measured in 2020 and 2021. We chose to measure all traits as frequent as possible. As we focused on the temporal development and fitted smoother functions, the interval between measurements could vary to some degree without affecting the outcome. Measurements took place between 1 June and 17 September in 2020 and between 18 February and 19 October in 2021.
Data exclusions	Root data from one monolith was excluded because roots at the tube surface were very scarce for unknown reasons.
Reproducibility	We used a relatively high sample size of 8 for the chamber experiment (8 monoliths per chamber) and a sample size of 5 for the bigger field plots, which should assure reproducibility in this system. Given the clear results, we believe a repetition of the experiment would yield the same outcome. Our description of the study allows for repetition, for example if other researchers wish to apply our methods to different ecosystems.
Randomization	The 16 monoliths were randomly assigned to one of the two chambers (initially by random sampling of the numbers 1 to 16 using the statistical programming language R) but the assignment was slightly modified to ensure both groups contained the same species with similar abundance.
Blinding	Monoliths were numbered and positioned in a random way in the field, such that the treatment each monolith belonged to was not obvious. In the climate chambers, blinding was not possible, because each climate chamber was assigned to a separate treatment. However, we do not expect that our measurements are prone to human bias, as photographs of canopy and roots were analysed computationally. Leaf lengths and other traits of individuals were measured in a standardized way by the same person to ensure consistency.

Did the study involve field work? ☒ Yes ☐ No

Field work, collection and transport

Field conditions	The field site is located in the alpine zone above the treeline, characterized by a short snowfree period of usually ca. 3 months (June/July-September). Air temperature during the growing season is usually between 5–15 °C, precipitation amounts to 300–400 mm (>1400 mm annually). More details are stated in the manuscript.
Location	Botanical Institute of the University of Basel, Switzerland (climate chambers) and ALPFOR research station at Furka pass (2440 m a.s.l., 46.577°N, 8.421°E), Switzerland.
Access & import/export	The investigated vegetation was accessible in the vicinity of the research station ALPFOR in Switzerland. No samples had to be imported/exported.
Disturbance	We excavated 16 monoliths of alpine vegetation (each 28 cm diameter) from the study site. The remaining holes were refilled with soil and vegetation to minimize visible effects of vegetation collection.

Reporting for specific materials, systems and methods

We require information from authors about some types of materials, experimental systems and methods used in many studies. Here, indicate whether each material, system or method listed is relevant to your study. If you are not sure if a list item applies to your research, read the appropriate section before selecting a response.

Materials & experimental systems

n/a	Involved in the study
☒	☐ Antibodies
☒	☐ Eukaryotic cell lines
☒	☐ Palaeontology and archaeology
☒	☐ Animals and other organisms
☒	☐ Clinical data
☒	☐ Dual use research of concern

Methods

n/a	Involved in the study
☒	☐ ChIP-seq
☒	☐ Flow cytometry
☒	☐ MRI-based neuroimaging
